# Comparative evaluation of electrode configuration for optimizing positive dielectrophoresis trapping of CCRF-CEM cells

**DOI:** 10.2142/biophysico.bppb-v23.0006

**Published:** 2026-02-17

**Authors:** Miftakhul Firdhaus, Ulya Farahdina, Nasori Nasori, Endarko Endarko, Agus Rubiyanto

**Affiliations:** 1 Laboratory of Medical Physics and Biophysics, Department of Physics, Faculty of Science and Data Analytic, Institut Teknologi Sepuluh Nopember, Surabaya, East Java 60111, Indonesia

**Keywords:** dielectrophoresis, CCRF-CEM, electrode geometry, positive dielectrophoresis, interdigitated electrodes

## Abstract

Dielectrophoresis (DEP) is a promising label-free technique for bioparticle manipulation, offering significant potential for diagnostic application such as isolation and trapping of cancerous CCRF-CEM cells. The efficacy of DEP trapping is critically dependent on the interaction between the cell’s dielectric properties and the spatial gradient of the squared electric field (∇|E^2^|), which is governed by electrode geometry. This study conducts a comparative evaluation, via finite element method (FEM) analysis, to quantify the positive dielectrophoresis (pDEP) force exerted on CCRF-CEM cells. Four distinct electrode configurations were analyzed: parallel (rectangular, triangular, cylindrical) and interdigitated. Based on a single-shell model for CCRF-CEM cells within a low-conductivity buffer, the Clausius-Mossotti factor was determined at 4.6 MHz, confirming a strong pDEP response that attracts cells to high-field regions. Results demonstrate that the pDEP force scales quadratically with applied voltage and is significantly enhanced by reducing the electrode gap. At a standardized 25 Vpp and 100 μm gap, the interdigitated configuration generated the highest maximum pDEP force, substantially exceeding the parallel rectangular, triangular, and cylindrical designs. Furthermore, the interdigitated geometry produced the most extensive and uniform high-force zones along the electrode edges, creating a superior trapping area. This comparative evaluation provides quantitative guidelines for optimizing electrode design, identifying the interdigitated configuration as the most effective for developing high-efficiency microdevices for CCRF-CEM cell trapping.

## Significance

This study bridges the gap between electrostatic theory and biophysical application for leukemia cell isolation. By integrating finite element analysis with a single-shell cell model, we demonstrated that interdigitated electrodes utilize a high-capacity ‘line trapping’ mechanism, significantly outperforming standard geometries for capturing CCRF-CEM cells. Crucially, we resolved the perceived trade-off between trapping force and cell safety, proving via the AC Schwan equation that high-voltage operation (25 Vpp) remains within the reversible electroporation threshold. These findings provide a quantitative framework for optimizing microfluidic devices dedicated to high-efficiency liquid biopsy.

## Introduction

Dielectrophoresis has emerged as a powerful technique for manipulating micro- and nanoparticles suspended in fluidic media. Its versatility has led to widespread applications in bioelectromechanics, environmental monitoring, and medical diagnostics, where target entities range from inorganic particulates to biological cells and bacteria [1]. One major advantage of DEP lies in its label-free operation, allowing for selective separation and trapping of particles without requiring chemical markers [2]. Fundamentally, DEP describes the motion of electrically neutral, yet polarizable, particles under a non-uniform electric field, driven by the interaction between the induced dipole moment and the spatial gradient of the electric field [3].

**Figure 1:**
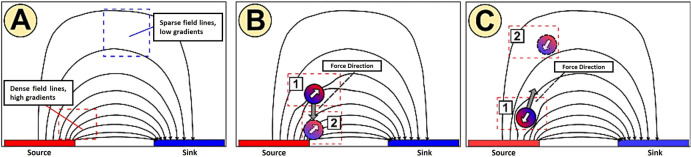
(A) Representation of electric field distribution: closely spaced field lines indicate a high electric field gradient, while widely spaced lines indicate a low electric field gradient. (B) Illustration of pDEP, where the particle migrates toward the high electric field region. (C) Illustration of nDEP, where the particle is repelled toward the low electric field region [3].

Depending on the particle and medium properties, two modes of DEP can occur: positive DEP (pDEP), in which particles migrate toward regions of high field intensity, and negative DEP (nDEP), where particles move toward regions of low field intensity (see Figure 1) [3]. The magnitude and direction of this force are primarily determined by the spatial distribution of the electric field, especially the squared electric field gradient $\left(\nabla \left|E^{2}\right|\right)$, which governs the strength of particle–field interaction.

**Figure 2:**
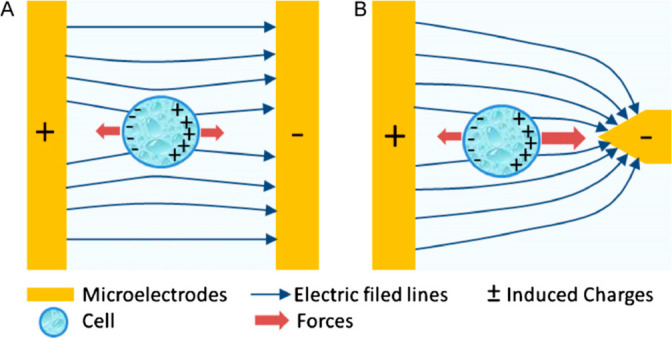
(A) Particle movement in a uniform electric field (electrophoresis), (B) Particle movement in a non-uniform electric field (dielectrophoresis) [4].

Various electrode geometries have been proposed to generate the required non-uniform electric field. Unlike electrophoresis, which employs uniform parallel-plate electrodes (Figure 2A), DEP utilizes non-uniform configurations such as parallel, triangular, cylindrical, or interdigitated electrodes (Figure 2B) [4]. Each geometry produces a distinct $\nabla \left|E^{2}\right|$ profile, thereby influencing the trapping efficiency, selectivity, and overall device performance [5]. Consequently, accurate modeling and simulation of these electric field patterns are thus essential before fabrication and experimental validation.

Several studies have investigated DEP electrode designs for trapping and separation applications. Hölzel et al. (2005) used triangular parallel electrodes with a 500 nm gap and 10 V input to trap single molecules, achieving $\nabla \left|E^{2}\right|$ ≈ 10^21^ V^2^/m^3^ [6]. Hyun et al. (2015) demonstrated DNA stretching using fiber-tip planar electrodes with 15 μm gap at 45 V [7], while Zhang et al. (2017) and Wang et al. (2014) explored cylindrical and interdigitated models, respectively, reporting gradients ranging from 10^14^ to 10^19^ V^2^/m^3^ [8]. Wang et al. (2014) compared flat interdigital and cylindrical electrodes, each with 10 μm gap and 1 V input. The interdigital setup reached 10^19^ V^2^/m^3^ but showed field instability down to 10^14^ V^2^/m^3^, whereas the cylindrical electrode maintained a more uniform field at approximately 10^17^ V^2^/m^3^ [9]. While these studies provide valuable insights, many have focused on analyzing the field gradient in general. However, there is a lack of systematic comparative analyses that directly relate these various electrode geometries to the actual DEP force experienced by specific biological cell targets, such as cancer cells. Understanding how these geometries quantitatively affect pDEP in CCRF-CEM cells is crucial for optimizing trapping efficiency in diagnostic applications.

Furthermore, in many developing regions, DEP device fabrication remains constrained by limited access to cleanroom facilities and microfabrication tools. Therefore, understanding which electrode geometry can yield optimal $\nabla \left|E^{2}\right|$ with simple, planar [10], interdigitated [11], and such as parallel plates, triangles, and cylinders, have also demonstrated efficacy for various DEP applications [12].

To address these limitations, this study distinguishes itself by bridging the gap between electrostatic simulation and biophysical applications specifically for leukemia cell isolation. Unlike prior studies that focused on generic particles, we integrate FEM simulations with a single-shell dielectric model of CCRF-CEM cells to quantify the pDEP trapping force magnitude. Furthermore, we introduce a safety assessment framework using the AC Schwan equation to validate that high-efficiency trapping can be achieved without inducing irreversible electroporation. Through a rigorous comparison of four electrode configurations, this work elucidates the transition from low-capacity ‘point trapping’ to high-throughput ‘line trapping’ mechanisms, providing a comprehensive design framework for the development of safe and effective cancer cell isolation devices.

## Materials and methods

### Electrode geometry and design

**Figure 3:**
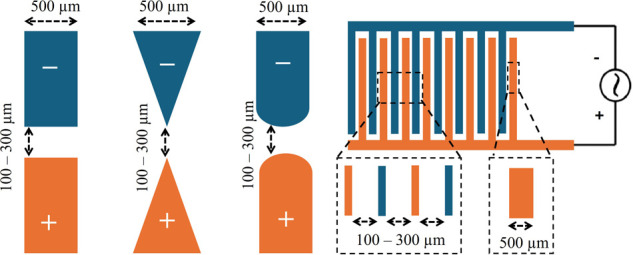
Geometry and dimensions of the four proposed electrode models. (from left to right) parallel rectangular, parallel triangular, parallel cylindrical, and interdigitated.

Four electrode configurations were modeled and simulated using COMSOL Multiphysics (Version 5.3) to represent commonly used DEP designs: (i) rectangular parallel, (ii) triangular parallel, (iii) cylindrical parallel, and (iv) interdigitated electrodes (Figure 3). Each model was constructed in a 2D symmetric top-view domain, representing a microfluidic channel with planar electrodes embedded on the substrate. The electrodes were designed with a width of 500 μm and thickness of 100 μm. Inter-electrode gaps of 100 μm, 200 μm, and 300 μm were considered to evaluate the effect of spacing on the electric field distribution.

### Boundary conditions and material properties

The electrostatic potential distribution within the computational domain was obtained by solving Laplace’s equation for a quasi-static electric field:

(1) $$\nabla^{2}V=0$$

where V is the scalar electric potential. The resulting electric field is expressed as:

(2) E=−∇V

The simulation medium was set to represent a low conductivity PBS with $\epsilon_{r}=80$ and σ=100mS/m. The electrodes were modelled as perfect conductors, with one electrode assigned a potential of +V/2 and the opposite electrode grounded at −V/2*.* All remaining boundaries were set as electrical insulation ($\hat{n}\cdot \vec{D}=0$). The applied voltages were varied between 5–25 Vpp at a fixed excitation frequency of 4.6 MHz. This frequency was specifically selected because it corresponds to the peak positive dielectrophoretic (pDEP) response of CCRF-CEM cells, maximizing the Re[fCM] and thus the trapping force.

### Simulation setup and meshing strategy

The Electrostatics (es) module under the frequency-domain study was employed to solve Laplace’s Equation (1) for electric potential distribution. Since the suspension medium is treated as electrically homogeneous and the system operates in the quasi-static regime, the spatial distribution of the electric field is determined exclusively by the electrode geometry and boundary potentials. A physics-controlled tetrahedral mesh was generated, with local refinement near electrode edges to accurately capture high-gradient regions. The minimum element size near sharp corners was set to 0.5 μm, and a mesh independence test was performed to ensure result convergence.

The squared electric field gradient was computed from the electric field vector components using:

(3) $$\nabla \left|E^{2}\right|=\nabla \left(E_{x}^{2}+E_{y}^{2}\right)$$

where $E_{x}$ and $E_{y}$ are the x- and y-components of the electric field, respectively.

### Biophysics model and DEP factor calculation

**Figure 4:**
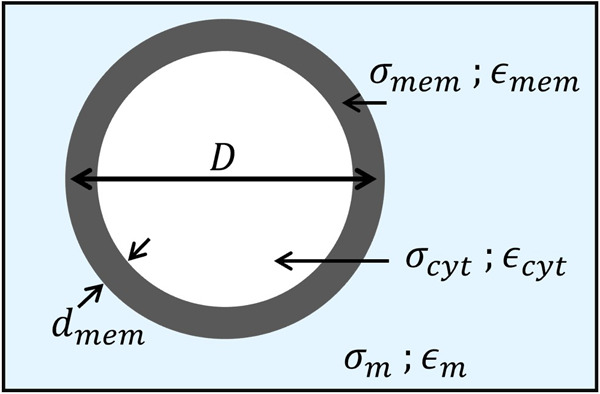
Single-shell model of CCRF-CEM cell used to determine the Clausius-Mossotti factor.

The Clausius-Mossotti factor (CM factor), which determines the DEP response was calculated using the equation:

(4) $$\kappa \left(\omega \right)=\frac{\epsilon_{p}^{*}-\epsilon_{f}^{*}}{\epsilon_{p}^{*}+2\epsilon_{f}^{*}}$$

where $\epsilon_{p}^{*}$ is complex permittivity of particle, and i is complex permittivity of suspension medium, $\epsilon^{*}=\epsilon -i\frac{\sigma }{\omega }$ where the value depends on the conductivity and angular frequency. To evaluate the CM factor, CCRF-CEM cells were modeled as spherical particles using a single-shell approximation, given that the dielectric signature at the operating frequency of 4.6 MHz is primarily dominated by plasma membrane. This model consists of cytoplasm encapsulated by a thin lipid membrane (Figure 4). The geometrical and electrical parameters of each cell type were taken from prior literature and are summarized in Table 1. The single-shell spherical model allows the CM factor to be reformulated in a simplified form (Equation 4), as described in [13],

(5) $$\epsilon_{p}^{*}=\epsilon_{mem}^{*}\left\{\frac{{\left[\frac{\left(r+d_{mem}\right)}{r}\right]}^{3}+2\left[{(\epsilon }_{cyt}^{*}-\epsilon_{mem}^{*})/\left(\epsilon_{cyt}^{*}+2\epsilon_{mem}^{*}\right)\right]}{{\left[\frac{\left(r+d_{mem}\right)}{r}\right]}^{3}-\left[\left(\epsilon_{cyt}^{*}-\epsilon_{mem}^{*}\right)/\left(\epsilon_{cyt}^{*}+2\epsilon_{mem}^{*}\right)\right]}\right\}$$

To achieve a pDEP response (Re[fCM]>0), a working frequency of 4.6 MHz was selected. At this frequency, (Re[fCM]) for CCRF-CEM cells reaches its maximum, producing the strongest DEP force on the target cells. Then, DEP force on the cell is calculated using equation:

(6) $$F_{DEP}=2\pi \epsilon_{m}r^{3}Re\left[\kappa \left(\omega \right)\right]\;\nabla \left|E^{2}\right|$$

where $\epsilon_{m}$ is the permittivity of the medium, r is the cell radius, Re[κ(ω)] is the CM factor, and $\nabla \left|E^{2}\right|$ is the squared gradient of the electric field obtained from the FEM simulation. The DEP force is strongly influenced by the particle volume $\left(F_{DEP}\propto r^{3}\right)$. Therefore, to evaluate the trapping selectivity, we calculated the DEP factor $\left(2\pi \epsilon_{m}r^{3}Re\left[\kappa \left(\omega \right)\right]\right)$ for CCRF-CEM compared to common blood contaminants (T-Cells and Red Blood Cells/RBCs). This comparison is important to ensure that the selected electrode configuration can preferentially trap target cells amidst a heterogeneous background cell population.

### Data extraction

**Figure 5:**
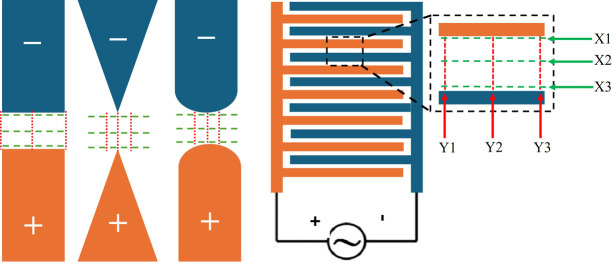
Illustration of data sampling locations for 1-dimensional plots of the squared electric field gradient across the 4 proposed electrode models.

Although ∇|E^2^| contour maps were generated as an intermediate step, the main analysis focused on the DEP force contour maps generated for each configuration. These DEP force maps were used to visualize the location and magnitude of trapping zones for CCRF-CEM cells. To obtain quantitative insight, one-dimensional data was extracted along specific line probes crossing the electrode gaps, as shown in Figure 5. For each configuration, three sampling points were defined along the x-axis (near the left electrode, mid-gap, and right electrode) and along the y-axis (bottom, midline, and top boundary). These line plots enabled a spatial comparison of DEP force profiles across different geometries and gap distances.

### Model validation

**Figure 6:**
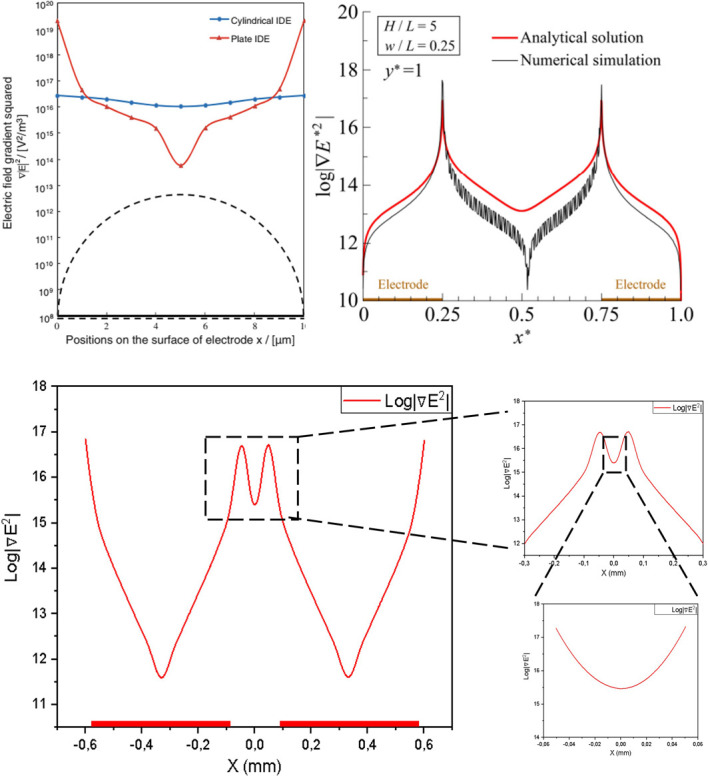
Validation of numerical setup. (A) Reference 1-dimensional profile of the $\nabla \left|E^{2}\right|$ at near the electrode, adapted from established studies [9,18], serving as benchmarks for the current simulation. (B) The corresponding profile obtained from the standarized simulation in this study. The simulation accurately reproduces the characteristic spatial distribution, featuring distinct symmetric peaks at the electrode edges (indicating high field localization) and a minimum in the mid-gap region. The close agreement in profile shape and trend confirms the reliability of numerical setup for subsequent force calculations.

To ensure the fidelity of the simulation framework prior to biophysical application, the numerical setup was validated by reproducing the electric field characteristics of interdigitated electrodes reported in established literature [9,18]. The validation focused on the $\nabla \left|E^{2}\right|$, as the dielectrophoretic force is linearly proportional to this parameter $\left(F_{DEP}\propto \nabla \left|E^{2}\right|\right)$. As shown in Figure 6, the simulated one-dimensional $\nabla \left|E^{2}\right|$ profile exhibits two distinct maxima at the electrode edges and a characteristic minimum in the mid-gap region. This spatial distribution demonstrates excellent congruence with the profiles reported by Wang et al. [9] and Nishikawa et al. [18]. The accurate reproduction of these sharp field gradients confirms the reliability of the Finite Element Method (FEM) setup, establishing a robust foundation for the subsequent calculation of on CCRF-CEM cells.

## Results

### Biophysical characterization and selectivity analysis

**Figure 7:**
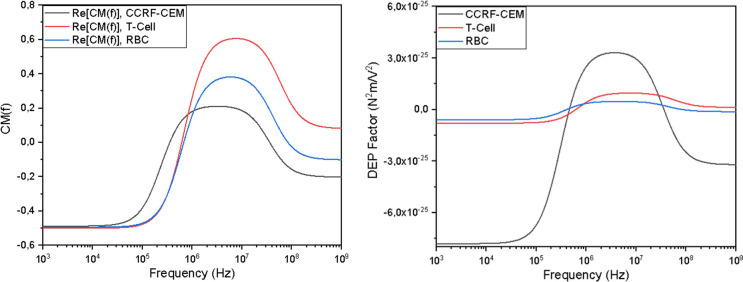
Frequency-dependent dielectric characterization and DEP force analysis of CCRF-CEM cancer cells relative to healthy blood cells (T-Cells and RBCs). (A) The real part of the Clausius-Mossotti factor Re[κ(ω)] as a function of frequency. All three cell types exhibit pDEP (Re[κ(ω)]>0) in the MHz range, indicating similar dielectric behavior. (B) The corresponding DEP Factor, calculated as $2\pi \varepsilon_{m}r^{3}Re\left[\kappa \left(\omega \right)\right]$, which accounts for particle volume. Despite the similar CM factors observed in (A), the larger radius of CCRF-CEM cells results in a significantly amplified force magnitude. At the operating frequency of 4.6 MHz, the pDEP force acting on CCRF-CEM cells is approximately 6-fold higher than that on T-Cells and RBCs, providing the physical basis for high-selectivity trapping via size-enhanced dielectrophoresis.

Prior to evaluating electrode geometries, the frequency-dependent dielectric response of the target cells was analyzed to determine the optimal operating conditions. Figure 7 presents the Clausius-Mossotti characterization (A) and the corresponding DEP force factor (B) for CCRF-CEM cancer cells compared to healthy blood cells (T-Cells and RBCs). As shown in Figure 7(A), the real part of the Clausius-Mossotti factor Re[κ(ω)] indicates that all three cell types exhibit positive dielectrophoresis (pDEP) in the MHz frequency range. Relying solely on dielectric properties would suggest difficulty in selective manipulation. However, when the cell volume is factored in $\left(F_{DEP}\propto r^{3}\right)$, a significant disparity emerges. Figure 7(B) reveals that at the operating frequency of 4.6 MHz, the DEP force factor for CCRF-CEM cells is approximately 6-fold higher than that of T-Cells and RBCs due to the larger radius of the cancer cells (6–8 μm) compared to blood cells (3–4 μm). This magnitude difference confirms that hydrodynamic drag forces can be tuned to elute the smaller healthy cells while the CCRF-CEM cells remain trapped by the significantly stronger pDEP forces. Consequently, all subsequent FEM simulations were conducted at 4.6 MHz to maximize this size-based trapping selectivity.

### Effect of applied voltage on trapping force

**Figure 8:**
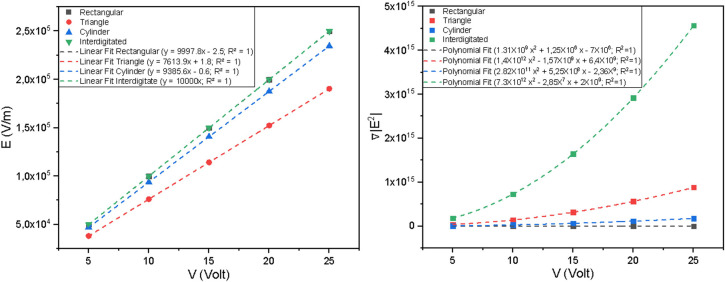
Relationship between the applied voltage and the resulting field parameters at a sampling point centered between the electrodes. (A) Peak electric field magnitude (E) and (B) squared electric field gradient $\left(\nabla \left|E^{2}\right|\right)$.

In the following sections, the simulation results are presented in terms of the squared electric field gradient (∇∣E∣2). Since the biophysical parameters of the cell model are constant, the magnitude of the DEP force is directly proportional to these gradient values. The magnitude of the pDEP trapping force is a critical parameter for overcoming fluid flow in microfluidic devices. Simulations were conducted with applied voltages ranging from 5 to 25 Vpp. The increase in voltage versus electric field was linear for all geometries (Figure 8a), while the trapping force applied to the CCRF-CEM cell showed a quadratic increase with applied voltage across all geometries (Figure 8b) with a regression value of R2=1 for both relationships, indicating perfect proportionality between the simulated values and theoretical expectations.

Quantitatively, the interdigitated electrode produced the highest $\nabla \left|E^{2}\right|$magnitude, reaching approximately $5.95\times 10^{15}{\;V}^{2}/m^{3}$ at 25 Vpp, followed by the rectangular ($3.5\times 10^{15}\;V^{2}/m^{3}$), triangular ($3.3\times 10^{15}\;V^{2}/m^{3}$), and last cylindrical ($1.9\times 10^{15}\;V^{2}/m^{3}$) configurations. This behavior aligns with earlier reports that interdigitated geometries concentrate field lines near electrode edges, generating stronger DEP forces [7]. The quadratic dependence of $\nabla \left|E^{2}\right|$ on voltage further corroborates that the dielectrophoretic force $F_{DEP}\propto \nabla {\left|E\right|}^{2}$ [19], highlighting voltage as a key control parameter for DEP actuation.

### Effect of electrode geometry on spatial trapping profiles

**Figure 9:**
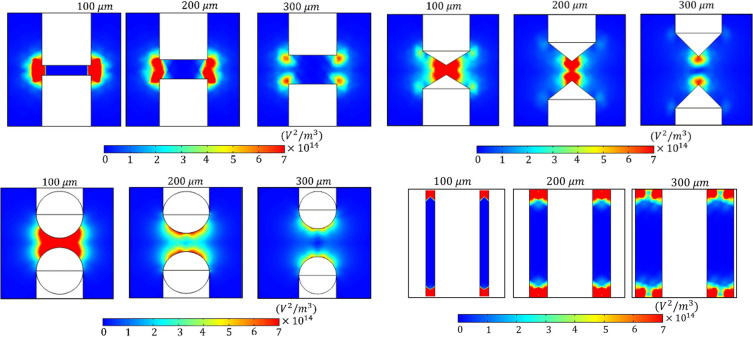
Comparative spatial distribution of the squared electric field gradient $\nabla \left|E^{2}\right|$ across four electrode geometries at 25 Vpp with gaps ranging from 100 to 300 μm. Since the DEP force is linearly proportional to this gradient $\left(F_{DEP}\propto \nabla \left|E^{2}\right|\right)$, these contour maps directly visualize the active trapping zones for CCRF-CEM cells. (A) Rectangular and (B) Triangular configuration exhibit high-intensity regions (red zones) strictly confined to sharp corners and tips, indicating a low-capacity ‘point trapping’ mechanism, (C) Cylindrical electrodes produces diffuse and weaker gradients due to the lack of geometric discontinuities, and (D) Interdigitated electrodes demonstrate superior distribution, with strong trapping gradients extending along the electrode edges, creating high-density array of capture zones suitable for high-throughput isolation.

**Figure 10:**
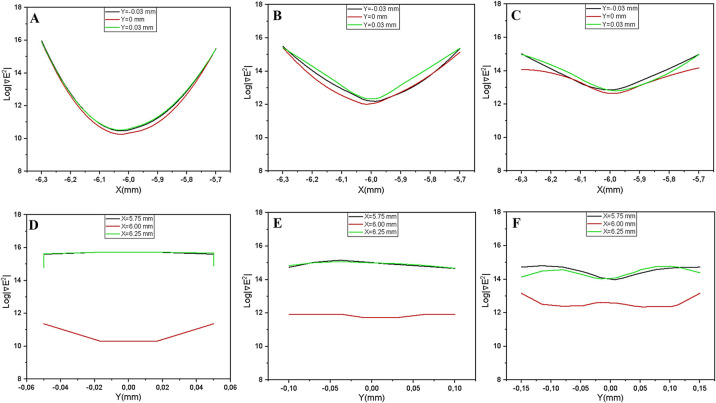
One-dimensional $\nabla \left|E^{2}\right|$ profiles for the rectangular parallel electrode configuration. (A-C) Profile extracted along x-axis for gap distances 100 μm, 200 μm, and 300 μm, respectively. (D-F) Corresponding profiles extracted along y-axis for the same distances. The sharp peaks observed near the electrodes indicate strong field localization. However, the gradient magnitude decays rapidly towards the gap center, confirming that effective trapping is restricted to the immediate vicinity of the electrode corners rather than the bulk fluid volume.

**Figure 11:**
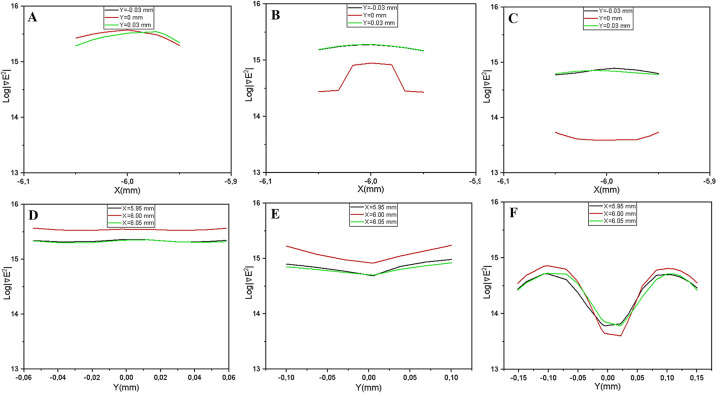
One-dimensional $\nabla \left|E^{2}\right|$ profiles for the parallel triangular electrode configuration (A-C) Profile extracted along x-axis for gap distances of 100 μm, 200 μm, and 300 μm, respectively. (D-F) Corresponding profiles extracted along y-axis for the same distances. The analysis reveals a strong dependence on electrode spacing. At a narrow gap of 100 μm (A and D), constructive field superposition between the opposing tips enhances the gradient magnitude near the center. However, as the gap widens to 200–300 μm (B, C, E, and F), the profile flattens significantly in the mid-region. This suggests that while triangular tips can focus on the field, the trapping stability at the gap center deteriorates with increasing separation distance.

**Figure 12:**
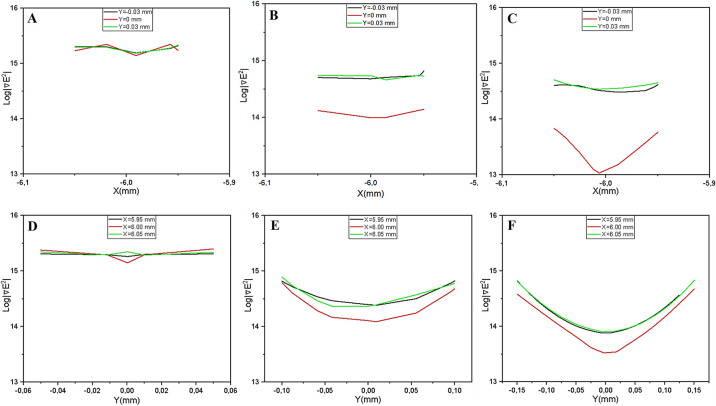
One-dimensional $\nabla \left|E^{2}\right|$ profiles for the parallel cylindrical electrode configuration. (A-C) Profile extracted along x-axis for gap distances of 100 μm, 200 μm, and 300 μm, respectively. (D-F) Corresponding profiles extracted along y-axis for the same distances. Unlike the planar designs, the cylindrical geometry produces smooth, symmetric gradient transitions without sharp discontinuities across all gap sizes. Consequently, the peak gradient magnitude is consistently lower than those of the rectangular and triangular models, resulting in weaker pDEP trapping forces that may be insufficient to retain cells against high hydrodynamic drag.

**Figure 13:**
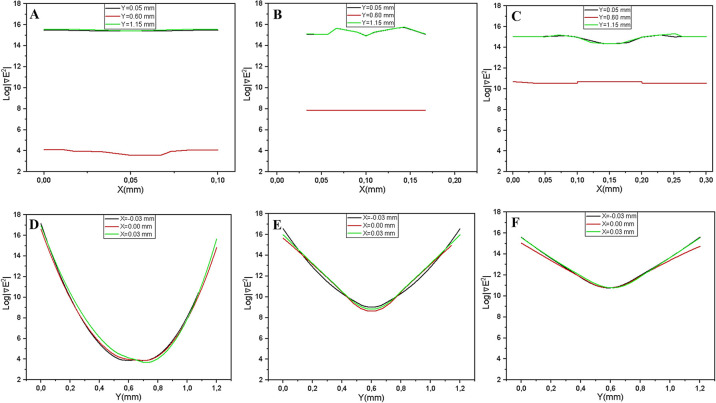
One-dimensional ∇|E2| profiles for the interdigitated electrode configuration. (A-C) Profile extracted along x-axis for gap distances of 100 μm, 200 μm, and 300 μm, respectively. (D-F) Corresponding profiles extracted along y-axis for the same distances. The profiles demonstrate the steepest spatial gradients among all investigated geometries, with force values dropping sharply from the electrode tips to the gap center. This pronounced variation creates a deep potential well that firmly locks cells at the electrode boundaries. Furthermore, the high-gradient profile is maintained even as the gap increases, indicating robust performance. The repetition of this profile across the digit array supports a high-capacity distributed trapping mechanism, validating the interdigitated design as the most effective geometry for maximizing cell capture.

To evaluate the efficacy of cellular capture, the spatial distribution of the pDEP force was analyzed across the four electrode geometries. Since the force magnitude is directly proportional to the squared electric field gradient $\nabla \left|E^{2}\right|$, the gradient contours presented in Figure 9 (2D map) and the extracted profile in figure 10–13 (1D plot) serve as a direct map of the active trapping zones for CCRF-CEM cells.

Effect of electrode gap on force amplification consistent with electrostatic theory, the electrode gap plays a decisive role in determining the magnitude of the trapping force. As observed in the 1D profile, narrowing the gap from 300 μm to 100 μm increased the peak force by approximately 2-fold across all designs. Crucially, as established in the viability analysis (section 3.4), this massive force gain at the 100 μm gap remains within the safe viability window $\left(V_{m}<1.0\;V\right)$ for CCRF-CEM. Consequently, minimizing the gap to 100 μm constitutes the optimal design strategy for maximizing retention.

Spatial confinement and trapping capacity, the electrode geometry fundamentally dictates the shape and capacity of the trapping zones, as revealed by the 1D gradient analysis:

1. Rectangular Configuration (point trapping): The 1D profile for the rectangular model reveals that $\nabla \left|E^{2}\right|$ peaks sharply near the electrode edges and decreases rapidly toward the gap center (Figure 10). This indicates strong field localization confined strictly to the corner tips. While these ‘hotspots’ generate high peak forces, the effective trapping area is extremely small (‘point trapping’), limiting the device’s capacity for high-throughput cell capture.

2. Triangular Configuration (Unstable Center): For the triangular model, the field distribution is highly sensitive to gap spacing. At smaller gaps (100 μm), constructive field superposition between opposing apexes shifts the maximum force toward the center. However, at wider gaps (300 μm), the profile flattens significantly in the mid-region (Figure 11). This creates a risk of unstable trapping where cells may not be firmly held against fluid drag in the center of the channel.

3. Cylindrical Configuration (Weak Trapping): The cylindrical electrodes exhibit the smoothest transitions, maintaining symmetric profiles across all gap sizes (Figure 12). However, the lack of sharp geometric discontinuities results in lower peak gradients compared to planar designs, translating to weaker holding forces that may be insufficient for robust cell retention.

4. Interdigitated Configuration (Optimal Line Trapping): The interdigitated electrodes demonstrate the most advantageous profile. The 1D analysis (Figure 13) shows the steepest spatial gradients, where force values drop drastically from the electrode tips $5.95\times 10^{15}\;V^{2}/m^{3}$ to the gap center $6.01\times 10^{4}\;V^{2}/m^{3}$. Unlike the localized points in the rectangular design, this high-force region extends along the entire length of the electrode digits (‘line trapping’). This significantly increases the active capture footprint, making the interdigitated geometry particularly suitable for high-efficiency focusing and trapping of large cell populations.

### Evaluation of electrode performance and biological safety

To determine the optimal configuration for CCRF-CEM isolation, a comprehensive evaluation was conducted integrating three critical metrics: (1) pDEP trapping force magnitude, (2) active trapping area (throughput potential), and (3) biological safety (cell viability). Table 2 synthesizes these performance indicators for the ‘worst-case’ operating condition.

Comparative performance and application insight based on the simulation results, the interdigitated geometry unequivocally outperforms the parallel electrode configurations. Quantitatively, it generates the highest peak trapping force (189 pN), providing superior retention against hydrodynamic drag compared to the weaker cylindrical and triangular designs. From a practical standpoint, spatial distribution is as critical as the force magnitude. Unlike the rectangular design which limits capture to low-capacity corner ‘points’, the interdigitated geometry utilizes a distributed ‘line trapping’ mechanism along the entire electrode array. This significantly expands the effective capture cross-section, maximizing the probability of retaining cells from a continuous fluid stream. This extended active area directly translates to higher trapping capacity and throughput, making the interdigitated design ideal for processing clinically relevant sample volumes where rare tumor cells must be isolated from large backgrounds.

To ensure that the trapping efficiency does not come at the cost of cell viability, the risk of electroporation was evaluated. The induced transmembrane voltage $\left(V_{m}\right)$ on the CCRF-CEM cells was calculated using Schwan’s equation in AC fields [20],

(7) $$V_{m}=\frac{1.5\;ER\cos\theta }{\sqrt{\left(1+{\left(\omega \tau \right)}^{2}\right)}}$$

Where, $V_{m}$ is transmembrane potential, E is electric field, R is cell radius, cosθ=1 due to spherical cell model, ω is angular frequency of AC voltage, and τ is time constant that can be written as

(8) $$\tau =RC_{m}\left(\rho_{cyt}+\rho_{f}/2\right)$$

Where, R is cell radius, $C_{m}$ is membrane capacitance per unit area (1 F/cm^2^) [21], $\rho_{cyt}$ is resistivity of cytoplasm 5.4 ohm m, $\rho_{f}$ is resistivity of medium 10 ohm m (derived from data in Table 1 and calculated using relation ρ=1/σ).

It is important to acknowledge that this analytical calculation assumes an idealized spherical cell with uniform membrane properties, whereas real biological cells exhibit surface heterogeneity and are exposed to highly non-uniform fields. To address this complexity, the safety assessment was explicitly conducted using the maximum electric field magnitude ($E_{\max}$) within the narrowest electrode gap (100 μm) at maximum voltage (25 Vpp), representing the ‘worst-case’ exposure scenario. As summarized in Table 2, even under these extreme conditions, the induced transmembrane potential for all geometries remained consistently below the critical irreversible electroporation threshold of 1.0 V [22]. Notably, the interdigitated configuration that generates the strongest trapping gradients induced a peak potential of only 0.287 V. Since induced potential scales with field strength, this result confirms that the cell remains within the safe, reversible regime troughput the entire trapping zone where the field strength is lower.

This finding is significant as it confirms that no trade-off is required between trapping force and cell viability. The device can be safely operated at the maximum input of 25 Vpp to maximize the pDEP force without compromising the structural integrity of the CCRF-CEM cells. This wide safety margin simplifies the operational protocol and enhances the device’s suitability for downstream biological analysis where cell viability is paramount.

### Study limitations

While this study establishes a rigorous comparative framework, several inherent assumptions must be acknowledged. First, the idealized 2D simulations assume a uniform electric field distribution along the z-axis, neglecting vertical gradients and wall effects. Consequently, 3D phenomena such as levitation are not captured. Second, the biophysical model employs a single-shell approximation and assumes perfectly spherical geometry. While sufficient for the frequency regime analyzed, it simplifies the intracellular complexity (e.g., nucleus) and surface heterogeneity (e.g., microvilli) characteristic of real biological cells. Finally, although trapping capacity is evaluated based on force magnitude and active area, coupled fluid dynamics simulations were not performed to model dynamic cell trajectories under continuous flow. Therefore, the reported force values should be interpreted as a theoretical performance ceiling and a representation of relative design trends, rather than absolute quantitative predictions of capture efficiency within a specific microfluidic environment.

## Conclusion

This study presented a comprehensive finite element analysis to optimize electrode geometry for the high-efficiency DEP trapping of CCRF-CEM cancer cells. By integrating biophysical cell modeling, the research established that at 4.6 MHz, the larger volume of CCRF-CEM cells results in a pDEP force factor approximately six-fold higher than that of background blood cells, confirming the feasibility of selective hydrodynamic separation. Among the evaluated geometries, the interdigitated configuration demonstrated superior performance, generating the highest peak trapping force at 25 Vpp and significantly outperforming parallel rectangular, triangular, and cylindrical designs. Furthermore, qualitative analysis confirmed that this configuration utilizes a ‘line trapping’ mechanism along the electrode edges, providing a substantially larger active capture area compared to the low-capacity ‘point trapping’ observed in rectangular electrodes.

Crucially, the biological safety assessment using Schwan’s equation confirmed that the proposed high-performance operating condition (25 Vpp with a 100 μm gap) does not compromise cell viability. The induced transmembrane potential remained consistently below the irreversible electroporation threshold (< 1.0 V) across all geometries. Consequently, the interdigitated electrode with a 100 μm gap is identified as the optimal design, offering a robust combination of high trapping force, high throughput capacity, and biological safety. These findings provide a validated design framework for the development of high-performance microfluidic platforms for leukemia cell isolation and liquid biopsy applications

Future research will prioritize experimental validation using ITO-based planar electrodes to verify the performance of the optimized Interdigitated design. Furthermore, subsequent studies will extend this computational framework to include three-dimensional field analysis and coupled fluid dynamics simulations, enabling the precise modeling of cell trajectories and trapping efficiency under realistic continuous flow conditions.

## Conflict of interest

All authors declare that they have no conflict of interest.

## Author contributions

AR and NN directed and supervised the research. MF and UF performed simulation, validation, and analysis data with the help of NN, EE and AR. MF and UF project administration. MF and NN visualize data and write drafts. All authors discussed and commented on the manuscript.

## Data availability

The evidence data generated and/or analyzed during the current study are available from the corresponding author on reasonable request.

## Acknowledgements

The authors gratefully acknowledge financial support provided by Institut Teknologi Sepuluh Nopember, which was provided through grant numbers 1898/PKS/ITS/2024 and 1179/PKS/ITS/2023. We also extend our appreciation to Indonesia Endowment Fund for Education Agency (LPDP) for scholarship support.

## Figures and Tables

**Table 1:** Parameters value of cells to obtain CM factor and DEP factor

Parameters	Unit	CCRF-CEM [14] [15]	T-Cell [16]	RBC [17]
**Size** Diameter Membrane Thickness	μm nm	14.2 10	8.4 10	6.2 4.5
**Cytoplasm** Relative Permittivity Conductivity	— S/m	40 0.185	103.9 0.65	59 0.31
**Membrane** Relative Permittivity Conductivity	— S/m	8 5×10^–7^	11.8 5×10^–7^	4.44 5×10^–7^

**Table 2:** Summary of simulated performance characteristics for CCRF-CEM trapping across each electrode configuration (applied voltage 25 Vpp, gap 100 μm)

Electrode Type	Max $F_{DEP}$* (pN)	Trapping Mode (Capacity)	Max Induced Potential** (V)	Viability Status	Overall Suitability
Parallel Rectangular	112	Point trapping (Low)	0.243	Safe	Moderate
Parallel Triangular	106	Unstable center	0.277	Safe	Low
Parallel Cylindrical	62.8	Smooth at edges (very low force)	0.233	Safe	Low
Interdigitated	189	Line trapping (High)	0.287	Safe	Excellent

* Force values are estimated based on the peak $\nabla |E^{2}$| and the calculated DEP factor for CCRF-CEM cells at 4.6 MHz. ** Values were derived using Schwan’s equation (eq. 7) with a cell radius 7.1 μm. The safety threshold for irreversible electroporation is ΔV<1.0V.
